# Progranulin Plays a Protective Role in Pneumococcal Meningitis by Inhibiting Pyroptosis

**DOI:** 10.1002/iid3.70140

**Published:** 2025-01-31

**Authors:** Jingyao Wang, Lihua Kang, Wenlong Xu, Jiangming Xiao, Yajun Min, Sijie Li, Changlong Zhou, Yibing Yin, Xuemei Zhang, Qun Zhang

**Affiliations:** ^1^ Department of Clinical Laboratory, Children's Hospital of Chongqing Medical University, National Clinical Research Center for Child Health and Disorders, Ministry of Education Key Laboratory of Child Development and Disorders, Chongqing Key Laboratory of Pediatric Metabolism and Inflammatory Diseases Chongqing Medical University Chongqing People's Republic of China; ^2^ Key Laboratory of Diagnostic Medicine Designated by the Ministry of Education, Department of Laboratory Medicine Chongqing Medical University Chongqing China; ^3^ Chengdu Women's and Children's Central Hospital, School of Medicine University of Electronic Science and Technology of China Chengdu China; ^4^ Department of Clinical Laboratory Women and Children's Hospital of Chongqing Medical University Chongqing People's Republic of China; ^5^ Department of Neurosurgery, Yongchuan Hospital Chongqing Medical University Chongqing People's Republic of China

**Keywords:** pneumococcal meningitis, progranulin, pyroptosis

## Abstract

**Objective:**

Pneumococcal meningitis is a serious infectious disease with a high mortality rate and a global presence, and survivors have different degrees of neurological sequelae as a consequence of the host response to the infection. Progranulin (PGRN) is a multifunctional autocrine growth factor that is also a major immunoregulator. We want to investigate the role for PGRN in Pneumococcal meningitis in vivo and in vitro.

**Method:**

Mouse and cell models were established to explore the protective effect and mechanism of PGRN against pneumococcal meningitis.

**Results:**

Progranulin plays a protective role in pneumococcal meningitis by inhibiting pyroptosis. Pyroptosis resulted from exposure of BV‐2 cells to the bacterium and this was confirmed in the in vivo model. Administration of the NLRP3 inflammasome inhibitor MCC950 to mice prior to infection inhibited pyroptosis and protected PGRN ‐/‐ mice and BV‐2 cell model from meningitis.

**Conclusion:**

This study implicates a protective role for PGRN in pneumococcal meningitis by inhibiting pyroptosis, indicating that PGRN may have therapeutic potential.

## Graphical abstract

Progranulin protect pneumococcal meningitis by inhibiting pyroptosis.

**Acronyms**
ASC: apoptosis‐associated speck‐like protein containing CARD
CNS: central nervous system
CSF: cerebrospinal fluid
ELISA: enzyme‐linked immunosorbent assay
GAPDH: glyceraldehyde‐3‐phosphate dehydrogenase
GSDMD: gasdermin D
NLRP3: NOD‐like receptor thermal protein domain associated protein 3
PGRN: progranulin
PM: pneumococcal meningitis
TNF‐α: tumor necrosis factor‐α
WT: wild type

## 1 Introduction

Bacterial meningitis is a leading cause of death related to infections worldwide and the most common and severe infectious disease of the central nervous system in children [1]. The introduction of vaccines has altered the profile for bacterial meningitis and currently *Streptococcus pneumoniae* is the most common cause for these disseminated infections [2]. Most importantly, the fatality rate for pneumococcal meningitis (PM) infections remains near 30% [3] and is the most prevalent cause of these types of infections.

PM patients suffer from varying degrees of neurological sequelae [4], including short‐term symptoms such as seizures, and long‐term symptoms such as as hearing loss, cognitive impairment, hydrocephalus, learning disability. The inherent characteristics of the pathogen and the corresponding host response can lead to specific brain lesions and clinical syndromes due to interference with nervous system functions. PM patents can suffer from long‐term nerve damage due to focal or diffuse axonal damage [5] and early axonal damage is difficult to diagnose through imaging or biochemical indicators [6]. Thus, it is very important to comprehensively understand the pathogenesis of PM and the corresponding host immune responses to enable more effective treatments.

The inflammatory response of brain is a double‐edged sword in the pathogenesis of bacterial meningitis. In the early stages of infection, neutrophil infiltration promotes the inflammatory response to control pathogen damage. In contrast, the presence of neutrophils during late infection stages can promote a robust endothelial cell reaction at the blood‐brain barrier that increases inflammation and ultimately leads to the destruction of the blood‐brain barrier structure resulting in severe nerve damage [7]. Thus, it is essential to comprehensively understand the regulatory mechanisms of the host inflammatory response to bacterial meningitis.

Progranulin (PGRN) is a growth factor that is a 68.5 kDa secreted protein and a multifaceted immune regulator. PGRN primarily acts via tumor necrosis factor receptor (TNFR) signaling and performs an important anti‐inflammatory role that has been demonstrated in multiple inflammatory models [8, 9]. In the nervous system, PGRN is primarily expressed and released by microglial and neuronal cells while astrocytes perform a PGRN storage function [10]. Mutations in the PGRN gene in humans are linked to tau‐negative, ubiquitin‐positive frontotemporal dementia [11] and Alzheimer's disease [12]. These mutations render the gene nonfunctional and the subsequent PGRN deficiency promotes neuroinflammation and neuron loss. Inflammatory disease of the central nervous system (CNS) is sometimes accompanied by upregulation of PGRN expression suggesting a role for this protein in the regulation of CNS inflammatory responses [13]. Interestingly, PGRN also performs anti‐inflammatory roles in infectious disease. In particular, PGRN is a TNFR antagonist and can inhibit production of pro‐inflammatory cytokines by direct binding [14]. However, whether PGRN functions in a similar manner during bacterial meningitis remains to be elucidated.

Our pre‐experiment demonstrated that PGRN levels are increased in mice and humans with bacterial meningitis. To investigate whether increased PGRN levels affect bacterial meningitis, we established pneumococcal meningitis in vivo and in vitro models to explore the possible mechanism of its influence on PM and evaluate the potential treatment of PGRN and MCC950 for PM.

## 2 Materials and Methods

### 2.1 Patients and Sample Collection

A total of 22 patients possessed cerebrospinal fluid positive cultures for bacterial meningitis, 12 with confirmed virus meningitis by analysis of cerebrospinal fluid (CSF) IgM as well as 10 non‐meningitis children were enrolled in the study. The age of non‐meningitis patients ranges from 0.5 to 7.5 years, while virus meningitis is 2 to 10, bacterial meningitis is 0.46 to 4. Non‐meningitis children were admitted to hospital with suspected meningitis but their routine examination of their cerebrospinal fluid and culture and biochemical results were all normal.

### 2.2 Mice and Experimental Protocols

C57BL/6 wild type (WT) and PGRN‐deficient (PGRN‐/‐) mice were purchased from Jackson Laboratory (Bar Harbor, ME, USA). The breeding and genotyping of PGRN‐/‐ mice followed the Jackson Laboratory protocol for these experiments and 8‐10 week old female mice were used for these experiments.

Mice were anesthetized and received intracerebroventricular injections of 15 μL suspension (4.5 × 10^6^ CFU) of *S. pneumoniae* serotype Ⅲ or an equal volume of saline for control mice as previously described [15, 16]. Injection of Evans blue confirms the modeling method. The common index was used to assess experimental PM infections as previously described. Brain tissue samples were excised under general anesthesia.

### 2.3 Cytokine and PGRN Quantification

Commercial ELISA kits were used to determine levels of PGRN (R&D Systems, Minneapolis, MN, USA) and IL‐6 and TNF‐a (Biolegend, San Diego, CA, USA) according to the manufacturer's instructions.

### 2.4 Clinical Scoring of Mice [15]

Loeffler's neurobehavioral five‐point system was used to evaluate the clinical symptoms of mouse meningitis: (5) normal motor activity and turned upright in < 5 s when put on their back; (4) decreased spontaneous activity, but still turned up in < 5 s; (3) turned up in > 5 s; (2) did not turn up; and (1) did not move or were in a coma.

### 2.5 Histology and Immunohistochemical Staining and Assessment

Consecutive 5 μm paraffin‐embedded brain sections were stained using haematoxylin & eosin (H&E) for routine morphological analysis by light microscopy. Immunostaining was performed on serial brain sections with 1:100 dilutions of rabbit anti‐PGRN polyclonal antibody (Proteintech, Rosemont, ILL, USA) followed by incubation with horseradish peroxidase‐labelled polymer anti‐rabbit secondary antibody (Boster Biological, Pleasanton, CA, USA). PGRN was evaluated with three independent observers that lacked any knowledge of the clinical and pathological data of the slides they were examining. The numbers of PGRN‐positive cells were counted from 10 representative microscopic fields and percentage of positive cells was calculated. The criteria used for assessment was previously reported as follows: < 5% of cell staining, negative; > 5% of cell staining, positive; positive staining was graded from weak/focal to moderate/focal or diffuse to strong diffuse.

### 2.6 Determination of LDH

LDH levels were determined using a commercial microplate kit (Nanjing Jiancheng Bioengineering Research Institute, Nanjing, China).

### 2.7 Cell Culture and Treatment

The mouse microglioma cell line BV‐2 was used to establish our cellular model and were obtained from Zhongqiaoxinzhou (Shanghai, China). Cells were seeded in 2 mL complete medium composed of minimal essential medium (MEM) containing 1% non‐essential amino acids, 10% fetal bovine serum, 1% penicillin/streptomycin (HyClone) and 1% sodium pyruvate in 6‐well plates at 7.5 × 10^5^ cells per well. *S. pneumoniae* serotype Ⅲ cells was added at a multiplicity of infection (MOI) of 40 per well and the plates were incubated for 6 h at 37°C. Supernatants and cell lysates were then collected for LDH tests and Western blot analysis.

### 2.8 Western Blot Analysis

Experimental mice were euthanized using 1.5% pentobarbital and the brains were perfused with normal saline from the left ventricle and then removed. Samples of brain tissue were lysed using 1 mL RIPA lysis buffer containing 1% PMSF to extract total protein. BV‐2 cells were lysed with 50 μL lysis buffer. Antibodies to the inflammasome components NLRP3, ASC, caspase‐1 and GSDMD as well as the cytokines IL‐1β and IL‐18 were obtained from Abcam (UK) and were used for Western blot analysis after protein electrotransfer.

### 2.9 Drugs

The inflammasome inhibitor MCC950 (Selleck, Houston, TX, USA) or PBS were injected at 20 mg/kg ip 24 h before bacterial injection [17].

### 2.10 Statistical Analysis

Differences in variables among groups were tested using Kruskal–Wallis test for the non‐normal distribution. Comparisons between human groups were examined using the Mann‐Whitney *U* test. AUC was used to evaluate the ability to discriminate bacterial meningitis from viral meningitis or control group. Spearman's rank correlation test was used to assess the correlations of CSF PGRN concentrations with nucleated leukocytes, glucose and albumin in CSF. Differences between mice were compared using the Student *t*‐test. Survival curves were compared using the log‐rank (Mantel–Cox) test. Brain homogenate supernatant LDH expression was compared using the unpaired t‐test and Western blot gray scale analysis was performed using Wilcoxon *U*‐test statistics. Significant differences are expressed by *p* values of < 0.05. Statistical analysis was performed with SPSS and Prism GraphPad [18].

## 3 Results

### 3.1 Comparison and Analysis of the Parameters in CSF and Blood From Control and Meningitis Group

As shown in Table 1, our study included 34 patients where 22 had diagnoses of bacterial meningitis and 12 with viral meningitis as well as 10 sex and age‐matched non‐meningitis control subjects. PGRN levels in plasma were dramatically elevated in bacterial meningitis patients compared with non‐meningitis controls (*p* < 0.05). Interestingly, we found no difference from the patients with viral meningitis. The PGRN levels in the CSF of viral meningitis patients were significantly higher than for the non‐meningitis controls (*p* < 0.01) while those for bacterial meningitis patients was even higher and significant (*p* < 0.001). PGRN concentrations in bacterial meningitis group were higher than that in the control group (*t* = 0.0018) (Table 1). The AUC of PGRN in CSF in the bacterial meningitis group is 0.727 (*p* = 0.003344, Table 2), which is relatively high. Interestingly, if we apply the blood routine parameters to logistic regression analysis, the AUC of the combination of PGRN, PCT, CRP, WBC, AUC will increase, the AUC of PGRN in blood combined with those parameters is 0.968, while the PGRN in CSF is 0.892 (*p* = 0.002, Table 3). The cutoff value, diagnostic specificity and sensitivity of PGRN in CSF in bacterial meningitis group are 2.170, 75.00, 81.82, in blood, they are 38.66, 66.67, 70.59 (Table 4). These results suggested that PGRN may play a role in bacterial meningitis and has potential for diagnosis.

**Table 1:** Patients demographics and clinical characteristics from two meningitis groups and non‐meningitis group.

General information	Non meningitis	Viral meningitis	Bacterial meningitis	Kruskal–Wallis *χ* ^2^	*p*
Number (boy/girl)	10 (4/6)	12 (8/4)	22 (12/10)		
Age (years)	4.0 (0.5–7.5)	3 (2.0–10.0)	2 (0.46–4)		
Cell (×10^6^/L)	0 (0–5)	66 (8.5–183)	1020 (110–2340)	27.67	< 0.0001
Nucleated cells (×10^6^/L)	0 (0–4.3)	14 (5.5–47.0)	520 (80–1180)	29.61	< 0.0001
Microalbumin (g/L)	160.0 (118–278)	580.0 (245.0–2100)	2740 (535.0–4405)	14.60	0.0007
Glucose (mM)	3.2 (3.1–3.7)	3.7 (3.3–4.8)	1.1 (1–1.7)	28.26	< 0.0001
PGRN (ng/mL, CSF)	0.72 (0.3–1.34)	1.73 (0.61–11.9)*	6.95 (2.38–30.87)** ^/^ ***	12.64	0.0018
PGRN (ng/mL, Blood)	35.06 (21.84–50.78)	34.58 (11.17–80.97)	47.49 (29.56–67.67)	3.324	0.1897
PCT (ng/mL)	0.49 (0.18–0.81)	0.10 (0.10–0.10)	0.80 (0.13–4.03)	10.38	0.0056
CRP (mg/L)	8.00 (8.00–9.00)	8.00 (8.00–10.50)	50.00 (7.50–85.00)	6.281	0.0433
White blood cells(×10^6^/L)	5.67 (3.79–9.99)	6.86 (5.42–10.22)	14.63 (9.48–19.95)	12.37	0.0021

* *p* < 0.01 vs. Non meningitis

** *p* < 0.01 vs. Viral meningitis

*** *p* < 0.001 vs. Non meningitis.

**Table 2:** The AUC of the parameters in CSF and blood for diagnosis of bacterial meningitis.

Parameters	AUC	*p*	95% Confidence interval
Cell (×10^6^/L)	0.9511	< 0.0001	0.8899 to 1.012
Nucleated cells (×10^6^/L)	0.9749	< 0.0001	0.9366 to 1.013
Microalbumin (g/L)	0.7995	0.001218	0.6545 to 0.9445
Glucose (mM)	0.9799	< 0.0001	0.9462 to 1.014
PGRN (ng/mL, CSF)	0.727	0.003344	0.5969 to 0.9088
PGRN (ng/mL, Blood)	0.667	0.07658	0.5021 to 0.8900
PCT (ng/mL, Blood)	0.7731	0.02774	0.5811 to 0.9651
CRP (mg/L, Blood)	0.7276	0.01512	0.5402 to 0.9151
White blood cells(×10^6^/L, Blood)	0.8221	0.0005051	0.6853 to 0.9588

**Table 3:** The AUC of the combination of PGRN and blood routine parameters for diagnosis of bacterial meningitis.

Parameters	AUC	*p*	95% Confidence interval
PGRN (blood) + PCT	0.889	0.010	0.717 to 1.000
PGRN (blood) + PCT + CRP	0.968	0.002	0.890 to 1.000
PGRN (blood) + PCT + CRP + WBC	0.968	0.002	0.890 to 1.000
PGRN (CSF) + PCT	0.877	0.002	0.737 to 1.000
PGRN (CSF) + PCT + CRP	0.885	0.002	0.751 to 1.000
PGRN (CSF) + PCT + CRP + WBC	0.892	0.002	0.760 to 1.000

**Table 4:** The cutoff value, sensitivity, and specificity of parameters in CSF and blood for the diagnosis of bacterial meningitis.

Parameters	Cutoff value	Sensitivity	Specificity
Cell (×10^6^/L)	71.00	95.24	84.21
Nucleated cells (×10^6^/L)	43.50	95.24	89.47
Microalbumin (g/L)	740.0	71.43	84.21
Glucose (mM)	2.940	90.48	94.74
PGRN (ng/mL, CSF)	2.170	75.00	81.82
PGRN (ng/mL, Blood	38.66	66.67	70.59
PCT (ng/mL, Blood)	0.318	69.23	70.00
CRP (mg/L, Blood)	36.00	60.00	100.0
White blood cells(×10^6^/L, Blood)	7.885	90.48	73.68

### 3.2 PGRN Expression Increased in Brain and Plasma in the Pneumococcal Meningitis Mouse Model

**Figure 1:**
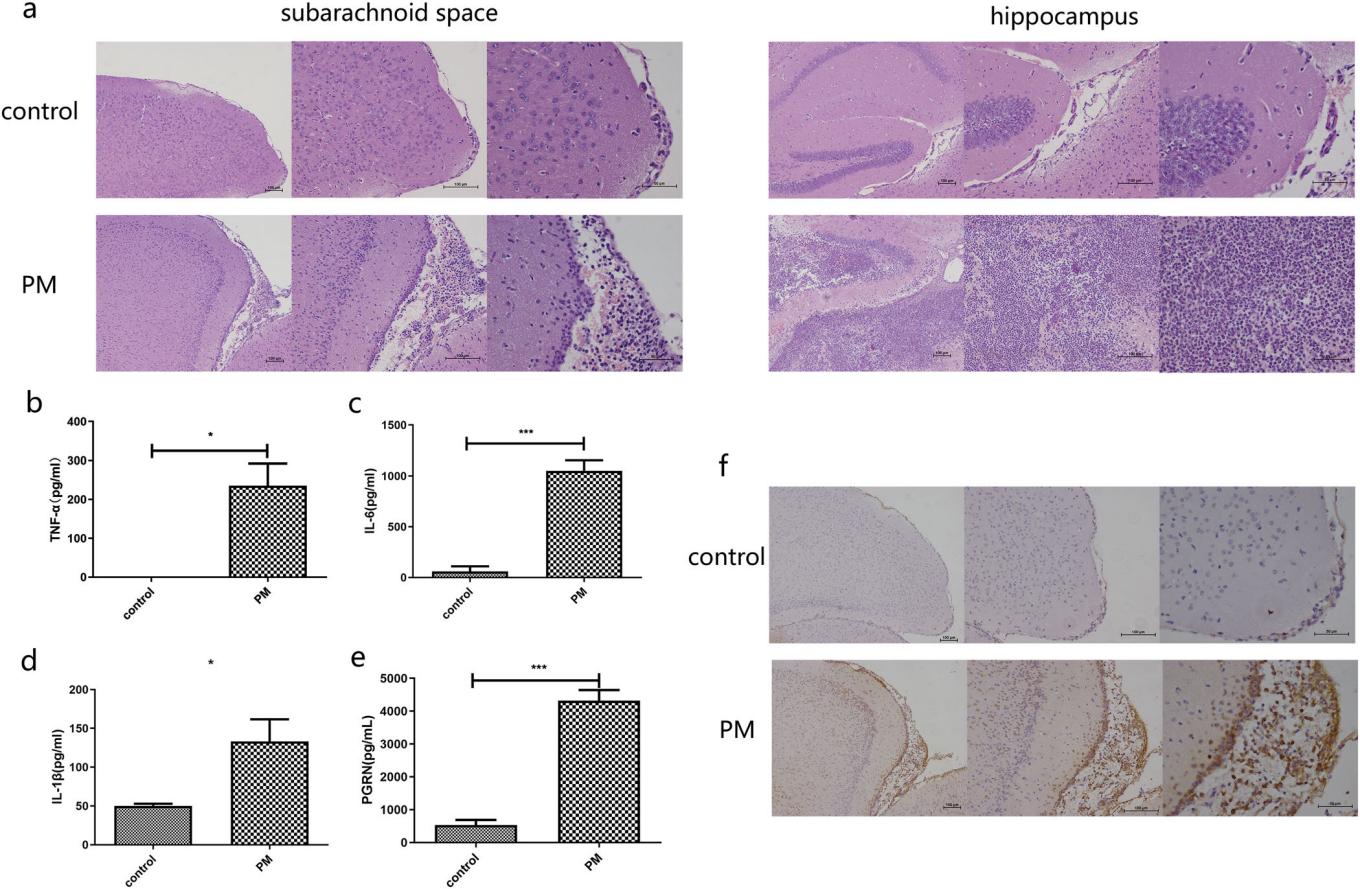
Histology and ELISA determinations of TNF‐α, IL‐1β, and IL‐6 in mice with experimental pneumococcal meningitis. (a) H&E staining of brain serial sections of the indicated mice (*n* = 4). (b) TNF‐α levels in brain homogenates at 24 h. (c) IL‐6 levels in plasma at 24 h. (d) IL‐1β expression in brain homogenates at 6 h. (e) Plasma levels of PGRN. (f) PGRN levels mice in brain serial sections (*n* = 4). Results of the above animal experiments are representative of three independent experiments.

We also utilized a mouse PM model that has been widely used for meningitis studies. We initially found that evans blue injected into the brains of mice was rapidly distributed throughout the CSF in mice with meningitis (Fig. S1). Serial sections of the brains indicated that mice with meningitis had more pronounced inflammatory infiltrates that was manifested by elevated neutrophil levels in the subarachnoid space and hippocampus compared with controls (Figure 1a). The serum of the PM mice also displayed significantly elevated levels of the inflammatory cytokines TNF‐α, IL‐6, and IL‐1β in serum and brain homogenates for the meningitis group (Figure 1b–d). These results are consistent with human meningitis, showing that we have successfully established a mouse model of pneumococcal meningitis.

When we examined PGRN levels in brain sections and serum samples from these mice, PGRN levels were dramatically increased for the meningitis groups (Figure 1e). Immunohistochemistry of brain sections also indicated that PGRN levels for the meningitis mice were greater than control mice (Figure 1f). PGRN expression in the brains of these mice was also consistent with our clinical findings and indicated the PM mouse model could also be used to study the role of PGRN in meningitis pathological processes. The presence of PGRN dampened the host immune response to pneumococcal meningitis.

**Figure 2:**
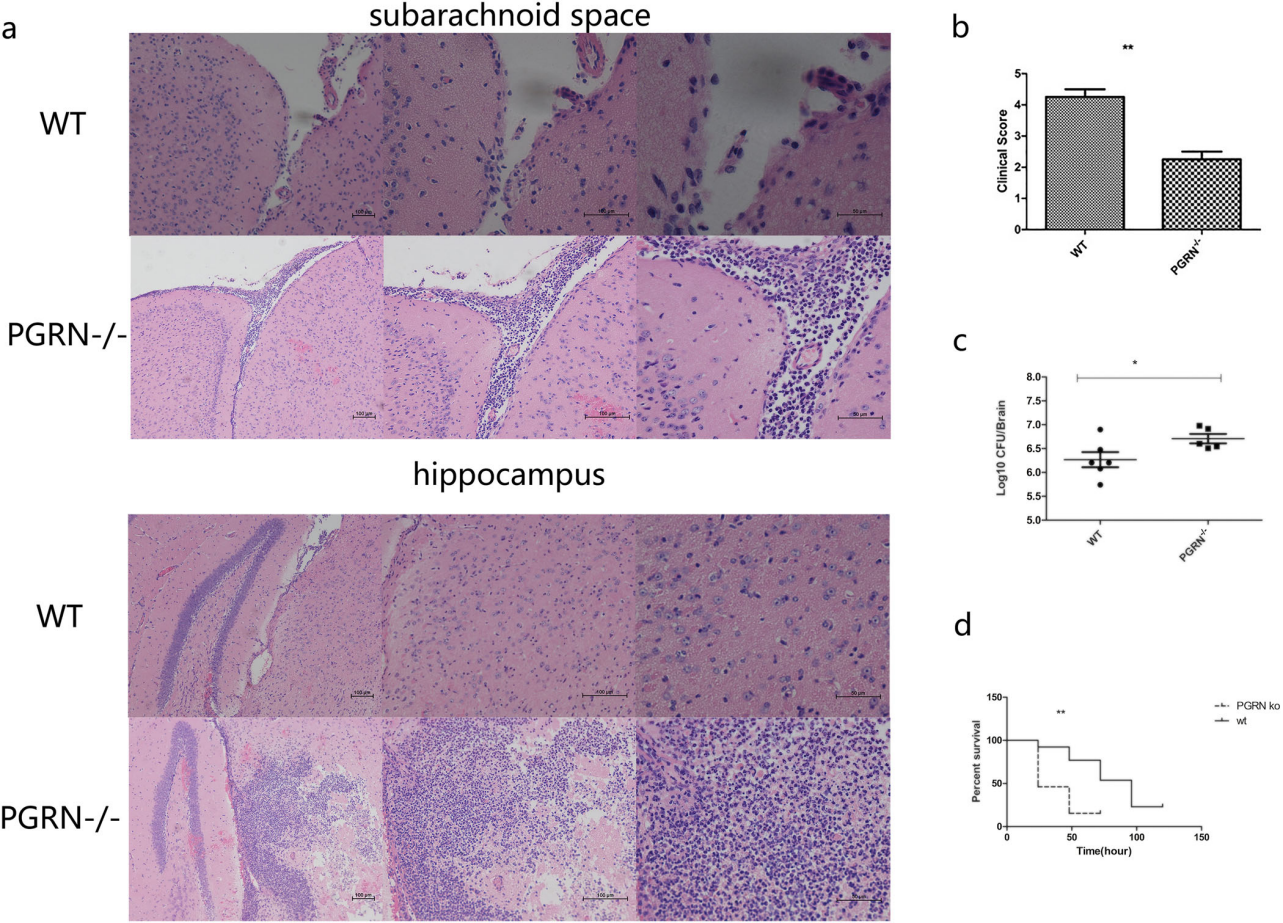
A comparison of WT and PGRN ‐/‐ mice with experimental pneumococcal meningitis. (a) H&E staining of brain tissue sections (*n* = 4). (b) Loeffler's neurobehavioral scores for mice with meningitis at 24 h postinfection (*n* = 4). (c) Bacterial loads in brains at 24 h (*n* = 6). (d) Survival curves for infected and control mice (*n* = 13). Results of the above animal experiments are representative of three independent experiments. *p < 0.05; **p < 0.01.

To clarify the role of PGRN in pneumococcal meningitis, we injected the standard strain of *S. pneumoniae* serotype III into the lateral ventricle of WT and PGRN ‐/‐ mice. The levels of brain inflammation at 24 h postinjection for the PGRN ‐/‐ PM mice were significantly higher than for WT. This was manifested as high levels of infiltrates around the subarachnoid space and hippocampus (Figure 2a). The Loeffler's neurobehavioral score for the meningitis group at 24 h indicated that clinical symptoms for the PGRN‐/‐ mice were significantly (*p* < 0.01) more serious than for the WT mice (Figure 2b). The bacterial loads in the brains of PGRN ‐/‐ PM mice were also higher than for their WT counterparts indicating the presence of PGRN is necessary for bacterial clearance in the brain (Figure 2c). The survival of PGRN‐/‐ mice was also reduced compared to WT (Figure 2d). These results indicated that PGRN is involved in the pathogenesis of pneumococcal meningitis and the protein exerts a protective role.

### 3.3 PGRN‐/‐ Pneumococcal Meningitis Mouse Brains Incur Enhanced Pyroptosis

**Figure 3:**
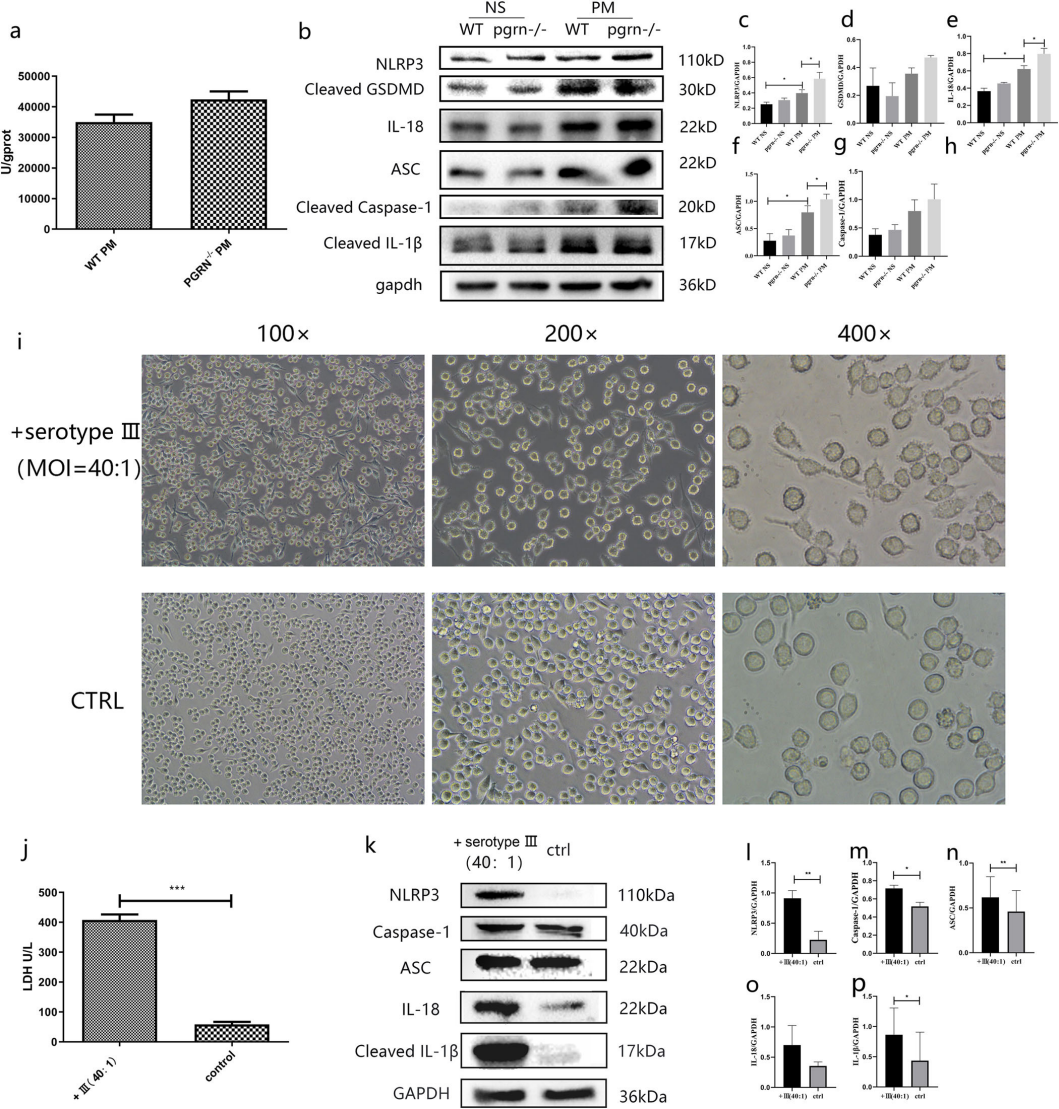
LDH and NLRP3 inflammasome components in brains of mice with pneumococcal meningitis and BV‐2 cells exposed to *Streptococcus pneumonia*. (a) NLRP3 inflammasome components and GAPDH (internal control) Western blots of brain tissue homogenates from saline control (NS) and PM mice (*n* = 3). (b–g) Quantification of Western blots using relative densitometry. (h–i) Morphology of BV‐2 cells exposed to *S. pneumoniae* serotype III and LDH expression in tissue culture supernatants. (j) Western blot detection of NLRP3 inflammasome components and controls as indicated in BV‐2 cells exposed to *S. pneumonia*. (k–o) Quantification of Western blot bands by densitometry (*n* = 3). Results of the above animal experiments are representative of three independent experiments. *p < 0.05; **p < 0.01, ***p < 0.001.

Previous studies had found that PGRN deficiency does not affect the antibacterial capacity of phagocytic cells. Therefore, the increased bacterial load we found in PGRN‐/‐ mice may be due to effects on phagocytic cell survival. Inflammasome pathway and pytoptosis markers in brain homogenates of WT PM mice were elevated for NLRP3, apoptosis‐associated speck‐like protein containing CARD(ASC), gasdermin D(GSDMD), caspase‐1, IL‐1β, and IL‐18 compared with the saline controls. Interestingly, these markers were elevated for the PGRN ‐/‐ PM mice compared with both these groups (Figure 3a–g). These results illustrated that pyroptosis had occurred in the brains of the PM mice and that PGRN ‐/‐ PM mice generated a more robust response indicative of a serious dysfunction in response to the infection. These data also revealed that PGRN plays a role in anti‐pyroptosis through the NLRP3 inflammasome.

### 3.4 NLRP3 and Pyroptosis Are Elevated in BV‐2 Cells Infected With *S. Pneumoniae*

In central nervous system diseases, NLRP3 expression in microglia is a proximal cause of pyroptosis [19]. We therefore wanted to establish whether the presence of *S. pneumoniae* induced phagocytic cell pyroptosis through NLRP3 and used a BV‐2 cell infection model for this purpose. BV‐2 cells exposed to the bacteria for 6 h were swollen compared with controls (Figure 3h) and LDH release into the cell medium was elevated compared with controls (Figure 3i) indicating membrane damage and suggesting that cell death may have occurred. Western blot analysis additionally indicated that caspase‐1, IL‐1β, and IL‐18 were all increased in the cells exposed to the bacteria, an indication that pyroptosis had occurred after coculture with *S. pneumoniae*. Consistent with these observations, NLRP3 and ASC levels were increased in the cocultured cells suggesting that pyroptosis was induced via activation of the NLRP3 inflammasome (Figure 3j–o).

### 3.5 MCC950 and PGRN Inhibited Pyroptosis in BV‐2 Cells Infected With *S. Pneumoniae*

**Figure 4:**
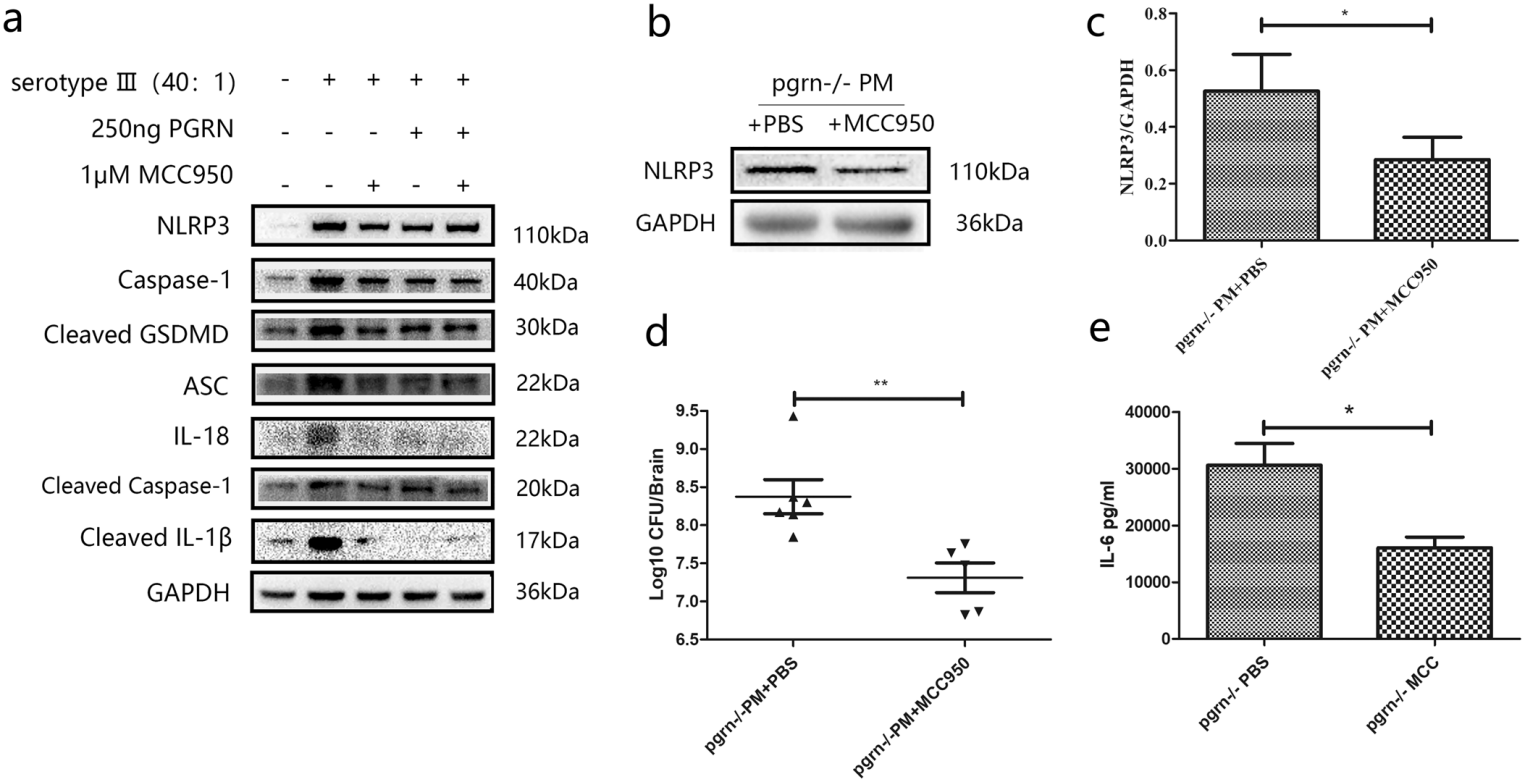
MCC950 prevent on BV‐2 cell model and PGRN‐/‐ pneumococcal meningitis in mice. (a) Western blot detection of inflammasome components and controls as indicated in BV‐2 cells exposed to *S. pneumoniae* treated by PGRN or MCC950. (b) NLRP3 expression in PGRN‐/‐ mice with experimental pneumococcal meningitis (PM) (*n* = 3). (c) Quantification of Western blot bands by relative densitometry of NLRP3 and GAPDH internal control (*n* = 3). (d) Bacterial loads in brains of infected and control mice (*n* = 6). (e) IL‐6 levels in brain homogenates of mice as indicated (*n* = 3). *p < 0.05; **p < 0.01.

We previously found a increased expression of NLRP3 and other pyroptosis related molecules in BV2 cells infected with *S. pneumoniae*, so we wanted to verify whether NLRP3 inhibitors (MCC950) can inhibit the expression of these molecules. The BV‐2 cells were pretreated with 1 μM MCC950 for 45 min before exposed to the bacteria, western blot analysis indicated that NLRP3, caspase‐1, GSDMD, ASC, IL‐1β, and IL‐18 were all reduced in the cells exposed to the bacteria (Figure 4), an indication that pyroptosis was inhibited by MCC950. Pyroptosis occurred in PGRN‐/‐ meningitis mice, suggested that PGRN may have a protective effect on pyroptosis, so we use recombinant PGRN protein to explore whether BV‐2 cells can inhibit pyroptosis after adding PGRN. The BV‐2 cells were pretreated with 250 ng PGRN for 45 min before exposed to the bacteria [20], western blot analysis showed that NLRP3, caspase‐1, GSDMD, ASC, IL‐1β, and IL‐18 were all reduced in the cells exposed to the *S. pneumonia* (Figure 4a), shows that PGRN also has the effect of inhibiting pyroptosis in BV‐2 cell model.

### 3.6 Inhibition of Inflammasome Activation Reduces Meningitis Symptoms in PGRN ‐/‐ Mice

Our results in the mouse and cellular models indicated that *S. pneumoniae* caused pyroptosis and that the presence of PGRN inhibited pyroptosis. However, a direct connection between PGRN and NLRP3 activation was lacking. To address this, we conducted our experiments in the presence and absence of the potent NLRP3 inhibitor MCC950. PGRN ‐/‐ PM mice receiving MCC950 displayed reduced NLRP3 levels in the brain (Figures 4b,c). When compared with the saline control group, the PGRN ‐/‐ PM mice treated with MCC950 displayed lower bacterial loads and IL‐6 levels (Figures 4d,e). This indicated that inhibiting the upstream inflammasome of the classical pathway of pyroptosis may promote *S. pneumoniae* clearance in the brains of PGRN ‐/‐ PM mice. This also suggested that PGRN inhibits NLRP3 activation to inhibit brain cell pyroptosis and thereby exerts a protective effect.

### 3.7 PGRN, Nucleated Leukocytes, and Glucose and Albumin Are Linked in the CSF of Meningitis Patients

**Figure 5:**
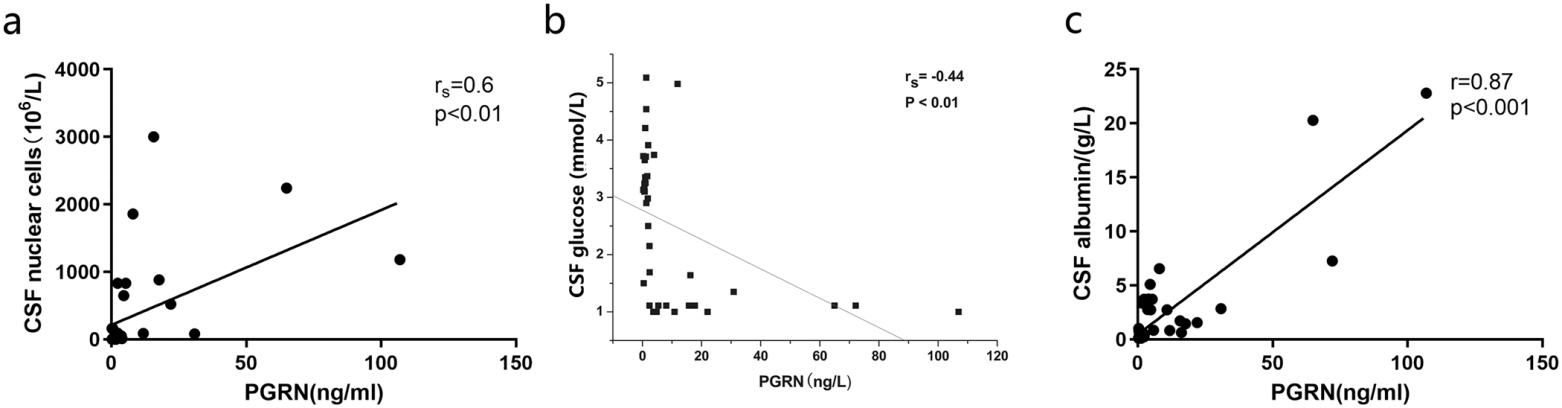
PGRN levels in CSF of patients with bacterial and viral meningitis are correlated with diagnostic markers of disease. (a) nucleated leukocytes (b) glucose and (c) albumin.

In addition to the direct culture of bacteria from CSF, the presence of nucleated leukocytes and lowered levels of glucose and albumin are important CSF markers used for diagnosis of bacterial meningitis. Unexpectedly, our bacterial meningitis patients possessed PGRN levels that were positively correlated with numbers of nucleated leukocytes and albumin levels and negatively correlated with glucose (Figure 5). Therefore, PGRN concentration in plasma and CSF could be used for the differential diagnosis of bacterial and viral meningitis.

## 4 Discussion

The use of vaccines over the last couple of decades has vastly changed the epidemiology of the bacterial meningitis, *S. pneumoniae*, group B streptococcus (GBS), and Neisseria meningitidis are the main causes of suppurative meningitis, survivors had similar neurological sequelae. Meningitis has a high mortality and serious sequelae but the pathogenic mechanisms are not clear. During microbial infections, pyroptosis is a type of inflammatory programmed death and this process attracts inflammatory cells to the infected site and inhibits the growth of bacteria by degrading invading pathogens. However, excessive pyroptosis will result in excessive release of cytokines and chemokines that mediate recruitment of immune cells from the peripheral circulation. This is especially true for neutrophils recruited to the site of infection. This cascade magnifies inflammation leading to pathology of the surrounding tissues and consequently to neurons. However, whether pyroptosis is related to the death of mice with meningitis has not been explored.

Recent studies have demonstrated that NLRP3 expressed in microglia may be a proximal cause of pyroptosis in central nervous system diseases [19]. NLRP3 recruits ASC that then aggregate with pro‐inflammatory caspases leading to the self‐activation of the latter and this promotes inflammation. The activation of caspases not only induces the production of inflammatory factors, but also triggers the pyroptosis effect. The key effector involved is GSDMD [21, 22]. Caspase‐1 can cleave GSDMD and release its active N‐terminus [23, 24] that binds to inner membrane lipids to form membrane pores causing local cell swelling, membrane rupture and leakage of IL‐1β, IL‐18, LDH and other cellular components [21]. Although IL‐1β and IL‐18 at low levels are required to maintain the normal function of the central nervous system, the lack of these cytokines leads to adverse consequences [25] while long‐term elevated levels are neurotoxic [26]. IL‐1β and IL‐18 can also trigger a cascade of secondary inflammation in the central nervous system that releases neurotoxic inflammatory mediators resulting in permanent neuroinflammation [27]. The results of our study indicated that in the meningitis mouse model constructed using *S. pneumoniae* serotype III, the NLRP3 inflammasome, ASC and caspase‐1, IL‐1β and IL‐18 expression all were increased. BV‐2 cell model show the same trend. LDH levels in the cell model culture supernatant increased significantly. Therefore, pneumococcal meningitis induced by S. pneumoniae may induce pyroptosis in brain by activating NLRP3. After use of MCC950, an inhibitor of NLRP3, the expression of NLRP3, GSDMD, ASC, Caspase‐1, IL‐18, IL‐1β were reduced, confirming that BV‐2 cell model occurred pyroptosis. The same situation occurred after the use of PGRN, indicating that PGRN can inhibit cell pytotosis. Interestingly, when the same dosage of PGRN and MCC950 were used at the same time in BV‐2 cells, the expression of these molecules did not appear to be lower, indicating that the dose and time of the combination of the two need to be further explored. Compared with the indicators of meningitis for WT mice, the protein markers in PGRN ‐/‐ meningitis mice were elevated suggesting that PGRN inhibits pyroptosis. At the same time, a large increase in the level of NLRP3 suggested that PGRN may exert this effect by inhibiting NLRP3. This was further verified by injection of the NLRP3 inhibitor MCC950 into the infected mice. The bacterial loads of mice pretreated with MCC950 were significantly lower than for controls and IL‐6 expression in brain homogenate supernatants were significantly reduced indicating MCC950 inhibited damage caused by PGRN deficiency. MCC950 has been shown to play a protective effect in other disease models. In mouse models of Parkinson's disease MCC950 prevented neuronal degeneration [28] and in cystic fibrosis it inhibited NLRP3 to reduce inflammation [29]. Our results suggested that MCC950 may have therapeutic potential in pneumococcal meningitis. In clinical treatment of meningitis, drugs often have difficulty crossing the blood‐brain barrier resulting in poor efficacy. To avoid the problem of drug permeability and timely treatment, intrathecal administration can also be used, but this increases suffering of patients [30]. Therefore, it is necessary to develop new drugs with high permeability and strong resistance. As a small molecule inhibitor, MCC950 has strong permeability and can freely pass through the blood‐brain barrier and has a short half‐life and low immunosuppressive effects. PGRN circulates in the cerebrospinal fluid and peripheral blood and can also reach the brain [31]. In familial frontotemporal degeneration and Alzheimer's disease, PGRN ‐/‐ mice showed increased expression of pro‐inflammatory factors, activation of microglia and increased differentiation of astrocytes. In contrast, overexpression of PGRN can reduce neuronal damage and memory loss indicating that PGRN has positive effect in most neurological diseases. In our study, PGRN CSF levels were positively correlated with the number of nucleated cells and protein content but negatively with glucose. These results suggested that PGRN in CSF was closely related to the laboratory index changes used to diagnose bacterial meningitis [32]. In children with bacterial meningitis, the expression of PGRN in the CSF and plasma were increased and was consistent with the results of other central system diseases [33].

## Author Contributions

Jingyao Wang and Lihua Kang were the main completers of the experiments, and analyzed the data. Jiangming Xiao, Wenlong Xu, Yajun Min, and Sijie Li assisted in specimen extraction during the experiment. Changlong Zhou provided the important experimental instrument of building the mice model. Yibing Yin, Xuemei Zhang, and Qun Zhang designed the topic and guided the experimental ideas.

## Ethics Statement

This study was conducted in accordance with the World Medical Association Declaration of Helsinki. This study protocol was reviewed and approved by the Ethics Committee of Chongqing Medical University, approval number is 2016(25). Human speciman have given their written informed consent to participate in this study.

## Conflicts of Interest

The authors declare no conflicts of interest.

## Supporting information

Supporting information.

## Data Availability

The data will be made publicly available without any restrictions. For further information, please contact the corresponding author Qun Zhang.
