# Supplementary material for: Progranulin Plays a Protective Role in Pneumococcal Meningitis by Inhibiting Pyroptosis
Source: Immun Inflamm Dis. 2025 Jan 31;13(2):e70140. doi: 10.1002/iid3.70140 (PMC11783684; doi:10.1002/iid3.70140)
Supplement: Supplementary file 1 — Supporting information. [file IID3-13-e70140-s001.docx]

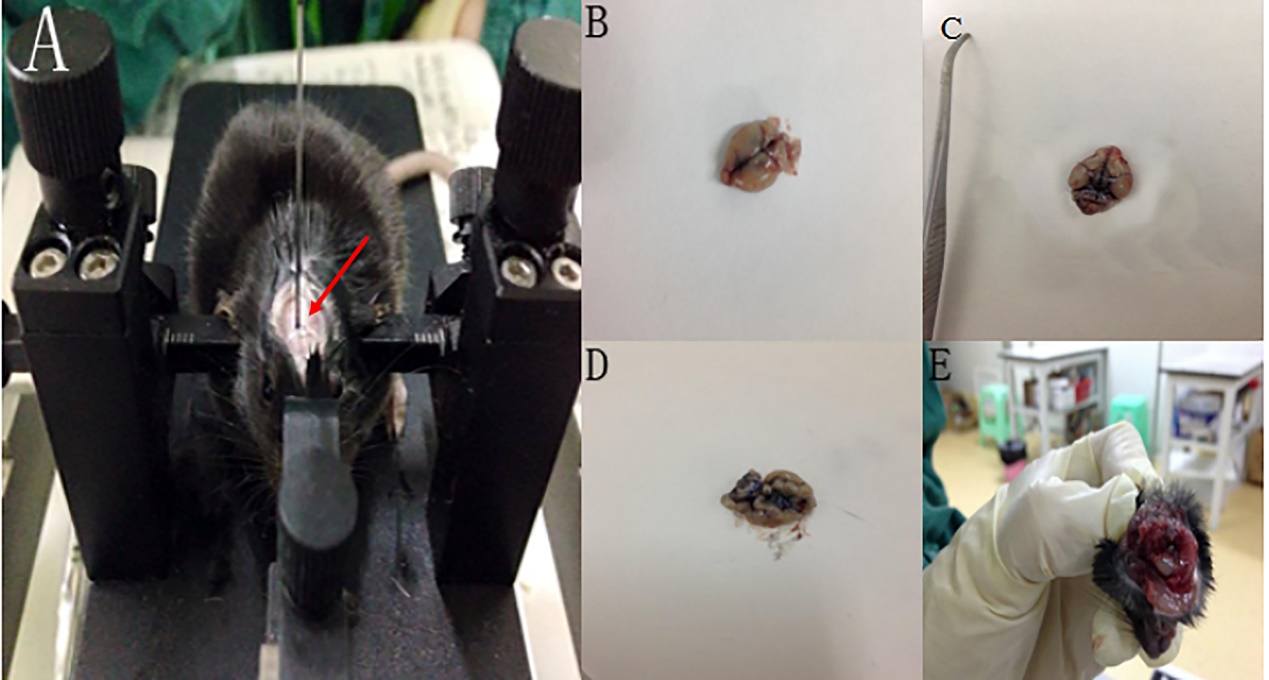


**Supplementary Fig 1. The brain anatomical diagrams after injecting blue dyestuff via intracerebral ventricular. (A) Injection location.** **After 2 minutes, dyestuff distributed throughout the CSF including basal spaces (B, C).**
